# Copper Effect on Microalgae: Toxicity and Bioremediation Strategies

**DOI:** 10.3390/toxics10090527

**Published:** 2022-09-06

**Authors:** Elena Cavalletti, Giovanna Romano, Fortunato Palma Esposito, Lucia Barra, Pasquale Chiaiese, Sergio Balzano, Angela Sardo

**Affiliations:** 1 Department of Ecosustainable Marine Biotechnology, Stazione Zoologica Anton Dohrn, Via Acton 55, 80133 Naples, Italy; 2 Department of Agricultural Sciences, University of Naples Federico II, Via Università 100, 80055 Portici, Italy; 3 Department of Marine Microbiology and Biogeochemistry (MMB), Netherland Institute for Sea Research (NIOZ), Landsdiep 4, 1793 AB Texel, The Netherlands; 4 Istituto di Scienze Applicate e Sistemi Intelligenti “Eduardo Caianiello” (ISASI), CNR, Via Campi Flegrei, 34, 80078 Pozzuoli, Italy

**Keywords:** microalgae, copper pollution, detrimental effects, reactive oxygen species, adsorption, heavy metal removal, bioremediation

## Abstract

Microalgae are increasingly recognised as suitable microorganisms for heavy metal (HM) removal, since they are able to adsorb them onto their cell wall and, in some cases, compartmentalise them inside organelles. However, at relatively high HM concentrations, they could also show signs of stress, such as organelle impairments and increased activities of antioxidant enzymes. The main aim of this review is to report on the mechanisms adopted by microalgae to counteract detrimental effects of high copper (Cu) concentrations, and on the microalgal potential for Cu bioremediation of aquatic environments. Studying the delicate balance between beneficial and detrimental effects of Cu on microalgae is of particular relevance as this metal is widely present in aquatic environments facing industrial discharges. This metal often induces chloroplast functioning impairment, generation of reactive oxygen species (ROS) and growth rate reduction in a dose-dependent manner. However, microalgae also possess proteins and small molecules with protective role against Cu and, in general, metal stress, which increase their resistance towards these pollutants. Our critical literature analysis reveals that microalgae can be suitable indicators of Cu pollution in aquatic environments, and could also be considered as components of eco-sustainable devices for HM bioremediation in association with other organisms.

## 1. Introduction

Heavy metals (HMs) are generally defined as elements with an atomic number greater than 20 and a density greater than 5 g cm^−3^ [1]. They are components of Earth’s crust and are naturally found at very low concentrations (from ppb up to 10 ppm) [2]. The main source of their occurrence in the environment, though, can be traced to anthropic activities, as they are broadly used in a wide variety of industrial applications, and they are present in fertilizers, pesticides and herbicides [2]. As a result, heavy metals can be released into the environment through industrial, civil or agricultural wastewaters [3], and since they cannot be degraded, they are considered persistent pollutants [4] and hazardous to humans [5] and ecosystems.

In aquatic ecosystems HMs can accumulate in sediments that act as a reservoir; upon their subsequent release due to various remobilization processes [6,7,8], those can be ingested or absorbed by living organisms and can build up along the trophic chain. This phenomenon—termed *biomagnification*—can lead to accumulation of HMs up to the higher trophic levels, posing a threat for both the environment and human health [9,10]. In some cases, high HMs concentrations have been found in liver and muscle tissues of several species of edible fishes [11,12,13,14]. Some known HMs accumulators are part of our diets, including the bivalve *Mytilus galloprovincialis,* a filter-feeder that has been used as a pollution indicator to assess levels of arsenic, cadmium (Cd), chromium, copper (Cu), mercury, manganese, nickel, lead (Pb) and zinc (Zn) in waters [15,16,17,18].

Some HMs, including cobalt, Cu, chromium, iron, manganese, molybdenum, nickel, selenium and zinc, are toxic to living organisms only if exceeding certain thresholds, while are considered essential at trace concentrations due to their known biological roles [19,20], while others, such as Cd, Pb and mercury, are regarded as biologically non-essential and exhibit toxic effects [21,22,23].

Cu, as an essential heavy metal, is involved in different biological activities thanks to its redox-active properties [24], due to its ready interconversion between two of its possible oxidation states, Cu^+^ and the more soluble Cu^2+^ ions. Being an essential co-factor for Cu-dependent enzymes, it is fundamental in biological processes such as energy metabolism (e.g., cytochrome C oxidase), antioxidative defence (e.g., Zn, Cu-superoxide dismutase) and iron metabolism (e.g., ceruloplasmin) [25].

Conversely, Cu levels above certain values can also be harmful due to its potential to catalyse the generation of toxic reactive oxygen species (ROS) [25]. Aside from acute Cu toxicity in humans [26], adverse health effects and impaired growth have been observed in both plants and animals [27] and in a wide range of living organisms.

In general, organisms showing variations in their level of abundance in a certain environment and alterations in their morphology, metabolism and/or genetic expression in consequence of the presence of certain contaminants, are often used as bioindicators for their environmental occurrence [28] and studied to assess their toxic effects. In aquatic environments, microalgae are one of the groups subjected to major HMs pollution exposure [29], and, being at the base of food webs in marine and freshwater ecosystems, represent an excellent candidate as HM indicator. Furthermore, some algal species, due to their ability to uptake HMs, are being investigated for their potential in bioremediation of HMs pollution [30,31,32]. Toward this aim, it is therefore imperative to investigate microalgae response to HMs stress. This review is aimed at describing the current knowledge of Cu effects on microalgae, including the main mechanisms of adsorption and compartmentalisation, its role in algal metabolism and negative effects on algal growth and physiology at high concentrations.

## 2. The Role of Cu as Essential Metal in Microalgal Metabolism

Cu plays a crucial role for living organisms that inhabit aquatic environments. Its history as an element essential to life traces back to the advent of oxygenic photosynthesis due to cyanobacteria metabolism, when a water-soluble ferrous iron, that until that moment had retained prominence in the catalysis of redox-involving biological processes, became oxidized to insoluble Fe(III) and lost its bioavailability, while oxidation of the insoluble, non-bioavailable Cu(I) led to soluble Cu(II) formation [33]. While Fe was then once again made accessible for organism metabolism through evolution of chelators and storage proteins such as ferritin, Cu also acquired a role in the exploitation of the oxidizing power of O_2_, due to its higher redox potential. Still, Fe and Cu metabolisms are often linked and the two metals are part of analogue enzymes involved in similar reactions and in some cases metabolic adaptation allows the organisms to use one catalyst over the other depending on metal availability [33,34,35]. Many algae and cyanobacteria synthesize two soluble proteins involved in electron transport both in photosynthesis and respiration, Fe-containing cytochrome C6 (Cyt) or Cu-containing plastocyanin (Pc), depending on Cu availability [35]. For example, in *Chlamydomonas*, under Cu-deficient conditions, Cu-sparing mechanisms involve the replacement of the more abundant Pc with Cyt, saving Cu for Cyt oxidase biosynthesis [36].

In phytoplankton, Cu is required for photosynthesis, respiration and defence against ROS via superoxide dismutase, and it is often a growth-limiting factor for these organisms [37]. In addition, in microalgae Cu is involved in transmembrane uptake of Fe [38,39] and may be vital for these organisms in Fe-limiting condition [40,41,42]. Exposure to Cu can also modify size, density and biochemical composition of algal cells. For example, in the green alga *Scenedesmus quadricauda* an increase in cell volume and in protein and carbohydrate content was observed in the presence of 1 µM of Cu, while a further increase (2.5 µM) of Cu concentration led to a decrease in cell abundance and chlorophyll-a content [43]. El Agawany et al., demonstrated a gradual increase in protein content of *Dunaliella tertiolecta* with low concentrations of three essential heavy metals, including Cu (ca. 79 µM), while increasing levels of heavy metals led to an inhibitory effect on protein synthesis with different grades [44]. Kong et al., found that, in the diatom *Thalassiosira oceanica*, Cu-limitation led to impaired photosynthetic efficiency due to decreased levels of Pc and rates of maximum photosynthetic electron transport and, consequently, to growth limitation. They also observed that Cu-limitation led to up-regulation of enzymes involved in fatty acid metabolism, which they argue could be directed at increasing turnover of lipids in the attempt to maintain membrane integrity in response to damage from oxidative stress [45]. The presence of Cu at sub-lethal concentrations also affected the photochemical and non-photochemical quenching in the freshwater microalga *Selenastrum gracile* with a consequent inactivation of reaction centres and damage to the photoprotection mechanisms, eventually affecting photosynthetic activity [46].

## 3. Mechanisms of Adsorption/Compartmentalization

As previously stated, Cu is an essential element for microalgal growth and metabolism required at nanomolar concentrations, but it can also be toxic at higher levels; thus, these organisms need to design strategies to tightly regulate its assimilation and homeostasis in the cytoplasm [47].

In general, in living organisms metal sequestration occurs in two steps: the first is usually fast and not dependent on cell metabolism, consisting in the adsorption of metal ions onto cell walls due to interactions between positive metal cations and negatively charged cell wall molecules; the second, considered the rate-limiting step, is slower and occurs only in living cells, where metal ions are actively transported across the cell membrane and enter into the cell via ion pores, channels or transporters [48,49,50]. In some cases, the mechanism involved in metal uptake is based on ions binding to multivalent ion carriers or, after binding to chelating proteins (e.g., MTs), entering the cell by endocytosis [51]. With regards to microalgae, the exact mechanisms responsible for metal uptake have not always been clearly elucidated. Quigg et al. [52] measured the rates of Cu accumulation for seven microalgal species showing a differential response of diverse algal species, although no information on the possible mechanism involved in Cu uptake was given. A very rapid HM cellular uptake has been observed in a cyanobacteria of *Synechoccus* sp., where a fast uptake took place within the first minute of Cu exposure, followed by a slower uptake in the next 15–20 min [52,53]. *Synechoccus* sp. has also been reported to be able to efflux internalized Cu, probably in attempt to maintain its homeostasis and counteract its toxicity [53].

Several Cu-transporters (CTR), permeases proteins present in the plasma membrane that move the ions in the cytoplasm, were identified in the model algal genus *Chlamydomonas*. CTRs are able to move Cu ions only in the +1 oxidation state, so their ability of metal compartmentalisation of Cu^2+^ ions is dependent on the activity of a cupric reductase [54].

To limit HMs toxic effects, some microalgae can produce compounds that complex with HMs outside the cell in order to reduce their bioavailability and entry in the cell; once inside the cell, some microalgae can extrude again HMs or internalize them into vesicles and/or organelles [34,55,56,57]. For example, *Skeletonema costatum* has been shown to sequester Cd^+2^ or Cu^+2^, administered in the culture media, in vacuoles to render the HMs less toxic, even though it has been observed that Cu was trapped to a lesser extent compared to Cd, resulting in greater growth inhibition and cellular damages [55]. In some cases, algae are able to remove very high Cu concentrations: the eustigamophycean *Nannochloropsis oculata*, for example, was able to completely accumulate up to 0.25 mM of Cu, but most of it was subsequently eliminated through metabolism [58].

## 4. Detrimental Effects of Cu at High Concentrations

In the presence of elevated Cu concentrations, microalgae are reportedly subjected to several detrimental effects, including reductions in growth rates, photosynthesis and respiration impairments, changes in cell and organelles size and morphology. In general, metal toxicity primarily results from metal binding to sulfhydryl groups of proteins resulting in the disruption of protein structure or displacement of an essential element [46,51].

Microalgal tolerance to HMs, HM stress markers and mechanisms activated to counteract negative effects, greatly vary between different species. Cu stress was found to induce membrane damages in *Chlorella sorokiniana* and *Scenedesmus acuminatus*, especially in the former, that exhibited a higher Cu accumulation and a lower antioxidants production, e.g., peroxidase (POX), ascorbate peroxidase (APX), glutathione reductase (GR) enzymes and proline, polyphenols and ascorbate (ASC) contents [59].

In the green microalga *Chlorella pyrenoidosa* Cu exposure caused significant changes in algal biomass, chlorophyll and carotenoids content [60]. Cu tolerance was also assessed in some diatom species. *Odontella mobiliensis*, for example, exhibited a reduction in growth rates and chlorophyll concentration within 72 h in a dose-dependent manner, an increase in cell size and impairment in cell structure [61]. Inhibition of cell division due to high concentration of uptaken Cu generates accumulation of photosynthetic products inside the cells through carbon fixation by photosynthesis, eventually resulting in enlarged cells [62].

Alterations in chlorophyll and carotenoid content after Cu exposure was also reported for two other marine species: *Chaetoceros calcitrans* and *Nitzchia closterium*. *C. calcitrans* was found to be the most sensitive one, showing also an increase in activity of antioxidant enzymes such as catalase (CAT) and APX [63]. Cu can also determine cytoplasmic and chloroplast vacuolization in algal cells, as demonstrated in the diatom *S. costatum*; this metal entered the cell and was partially located in spherical bodies found within the vacuoles [55].

Levy and co-workers [64] demonstrated that Cu sensitivity was mainly linked to the ability of internalise this metal inside cells and to detoxification mechanisms adopted by microalgae. They performed a study of several algal strains from different taxonomic groups, and did not find significant correlation among Cu detrimental effects and cell size or cell wall composition. Indeed, they found that both small and large microalgal species can be highly sensitive to Cu exposure, and that naked species (in this case the chlorophyte *D. tertiolecta*) can be more tolerant than diatoms (*Minutocellus polymorphus*) which possess a siliceous cell wall [64].

In another study, performed using one species of the genus *Desmodesmus* as test-organism, variations in the Cu removal efficiency, growth, ultrastructure and cellular metabolite content associated with Cu exposure as well as different pH were assessed [65]. That species was found to be able to grow in media supplemented with Cu, with a pH-dependent adsorption efficiency of the metal. Metabolic alterations associated to Cu exposure and medium acidity mostly affected sugars and amino acids content [65].

Overall, the above-mentioned studies suggest that Cu effects on microalgae can be detected in relatively short time, and pave the way for the employment of these microorganisms as sensors for Cu pollution in aquatic environments. Unfortunately, considering the huge variety of microalgae colonising freshwater and marine environments, the available literature gives information only on a limited number of species. Moreover, different experimental conditions and the diverse methods used for the detection of cell damages make difficult the detection of precise thresholds of Cu tolerance for test-organisms.

**Figure 1:**
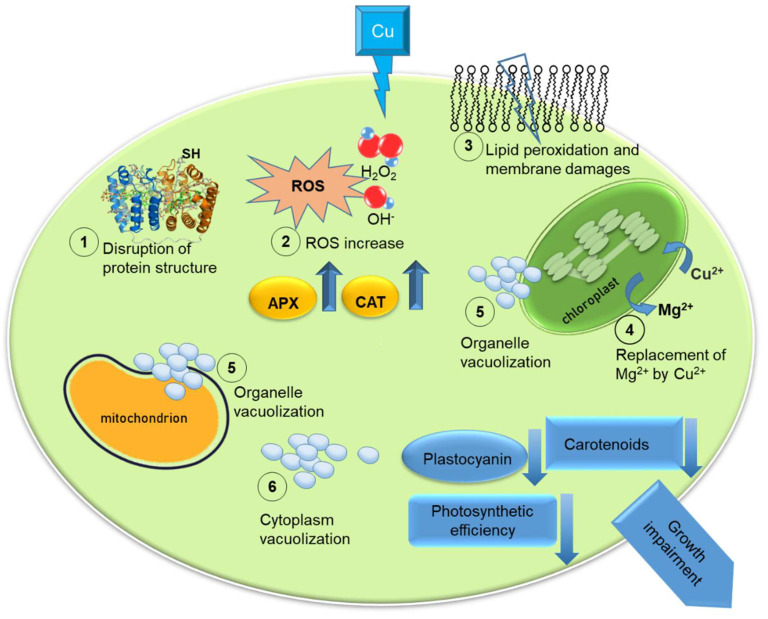
Illustration of the effects of high copper (Cu) concentrations on microalgal cells: disruption of protein structure (1), increase in reactive oxygen species (ROS) (2); lipid peroxidation and membrane damages (3); organelles vacuolization (4); replacement of Mg^2+^ by Cu^2+^ (5); cytoplasm vacuolization (6). Upward arrows indicate an increase in ascorbate peroxidase (APX) and catalase (CAT) in the cell; downward arrows indicate a decrease in availability of carotenoids and plastocyanin, and impairment of photosynthetic efficiency. The outcome of these effect is the impairment of growth rate.

In Figure 1, we illustrated the principal effects of Cu high concentrations on microalgal cells.

**Figure 2:**
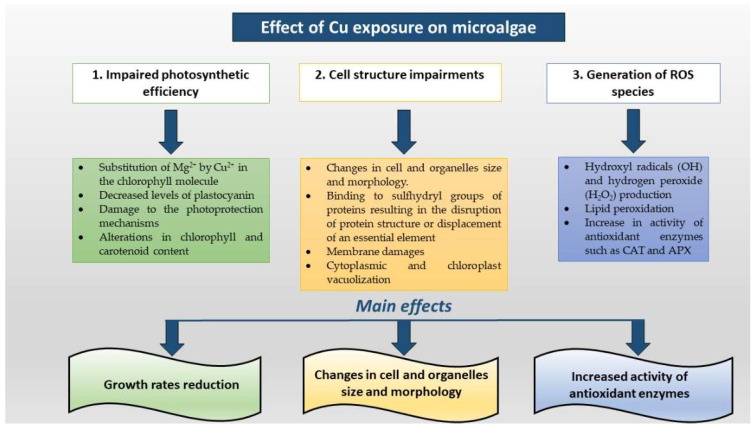
Detrimental effects of Cu on microalgal photosynthetic apparatus, on cell and organelle impairments and on the activity of antioxidant enzymes.

A schematic description of the main alterations of photosynthetic efficiency, shape modifications of cells and organelles, ROS generation is showed in Figure 2.

## 5. Generation of Reactive Oxygen Species

The inhibition of growth and photosynthetic processes in HM-stressed algae is often associated with the generation of reactive oxygen species (ROS) [66]. It is well known that ROS—such as superoxide hydroxyl radicals (OH) and hydrogen peroxide (H_2_O_2_)—are produced in cells when exposed to environmental stresses, e.g., exposure to high light intensities, UV radiation and heavy metals [67]. Microalgae have evolved numerous protective mechanisms that serve to scavenge ROS before they can severely damage sensitive parts of the cellular machinery. Defence against ROS includes low molecular mass compounds, such as glutathione (GSH), ascorbate, flavonoids, a-tocopherol and carotenoids, as well as enzymatic catalysts (e.g., CAT, superoxide dismutase and POX) [68,69].

It has been proposed that the chlorophyll-a reduction at higher Cu concentrations causes the inhibition of the synthesis of d-aminolevulinic acid and protochlorophyllide reductase, pigments disruption and membrane lipids peroxidation by ROS species. Cu pollution also inhibits chlorophyll integration into chloroplast photosynthetic membrane [62].

The role of reactive oxygen species (ROS) in Cu toxicity was assessed in two freshwater green algal species, *Pseudokirchneriella subcapitata* and *Chlorella vulgaris*. ROS concentration was found to be dependent upon irradiance and time, and caused impairments in photosynthetic activity and growth rates. However, effects of high Cu concentration on photosynthesis were different for the two species, leading to a slight reduction in photosynthetic activity in *P. subcapitata*, but not in *C. vulgaris*. These results indicate that these differences are likely due to diverse and species-specific ROS defence systems rather than to differences in the cellular ROS content [70]. The cellular defence mechanisms used by the marine diatom *Phaeodactylum tricornutum* to cope with Cu toxicity were investigated measuring the activity of the antioxidant enzymes such as superoxide dismutase (SOD) and CAT activity that increased in a few hours after Cu addition. Glutathione reductase (GR) activity, after an initial partial inhibition, was also enhanced, indicating the need to restore the oxidative balance of glutathione. Ascorbate peroxidase (APX) and exopolyphosphatase (PPX) activity was not significantly affected by Cu treatment, suggesting that in *P. tricornutum* CAT is the major enzyme for scavenging H_2_O_2_ [71].

In *Scendesmus vacuolatus*, exposure to increasing Cu concentrations induced an increase in protein and malondialdehyde (MDA) content, a measure of lipid peroxidation, and, at the highest Cu doses, a decrease in the chlorophyll-a/chlorophyll-b ratio, linked to an increase in CAT and SOD activities and GSH content. On the contrary, *Chlorella kessleri*, although able to survive only at lower Cu concentrations when compared to *S. vacuolatus*, did not show significant differences in the enzymatic and antioxidant activities when exposed to sub-lethal Cu doses, showing that also in this case the response to Cu is species specific [72].

As is well known, ROS production is also associated to other detrimental effects on microalgal cells. For example, the model species *C. vulgaris* exposed to different Cu sources—Cu oxide (CuO) nanoparticles (NPs), microparticles (MPs) and ions—underwent oxidative stress, metabolic alterations, which were similar for all Cu sources, and membrane impairments [73]. However, investigations on the specific response of microalgae to Cu in terms of ROS production and enzymatic pathways activation to cope with oxidant cellular stress is still poor. For this reason, experimental works that go in the direction of holistic approaches, such as an integration of transcriptomic, metabolomic analyses are necessary. A further step in this field could be the optimization of culture conditions (e.g., nutritional and physical parameters) to enhance the production of potent antioxidants able to scavenge ROS formation in HM-polluted environments.

## 6. Phytochelatins and Metallothioneins Protective Role against Heavy Metal Toxicity

Microalgae employ numerous strategies to counteract heavy metal toxicity, although the synthesis of thiol-containing peptides is considered the most specific and interesting. Indeed, cysteine-rich polypeptides play a key role in cell defence against oxidative and metal stress. The sulphide groups present in the side chain of cysteine residues possess a negative polarity resulting in electrochemical affinity towards HM cations; in addition, sulphide is a strong reductant able to react with reactive oxygen species protecting the cell against oxidative stress. Glutathione, the simplest cysteine-rich peptide, provides protection against oxidative stress [74,75] and has been occasionally reported to be released after exposure to HMs [76,77].

Phytochelatins (PCs) are polypeptides consisting of 2 to 10 GSH units and are enzymatically produced: GSH is converted to γ-glutamylcysteine which is, in turn, converted to PC [78,79,80]. PCs are known to bind HMs and to transport them within the cytosol towards the vacuole, [79,81,82]. In some cases, HMs can accumulate in chloroplasts and mitochondria, as observed for *Euglena gracilis* [83,84]. PCs are likely to occur in all photosynthetic organisms and genes coding for PC-biosynthetic enzymes have been detected in green algae and, at a minor extent, in diatoms, haptophytes and dinoflagellates [85,86]. Similarly to GSH, the cellular concentration of PCs has been reported to increase at increasing HM concentrations in diatoms, haptophytes and green algae [80,87,88]. High concentrations of GSH and γ-glutamylcysteine were found in the terrestrial green alga *Stichococcus minor* while growing in the presence of 10 µM Cu, suggesting a crucial role of PC precursors in Cu detoxification [89]. Zhang et al. [90] compared the release in PCs by *Chlorella* sp. after exposure to Cu, Cd and Pb and found the highest PC content in cultures spiked with Cd.

In contrast to GSH and PCs, metallothioneins (MTs) are genetically encoded proteins consisting of 40 to 160 amino acids, with <10% aromatic residues and a 15–35% cysteine content [91]. MTs are also known to bind HMs minimising their detrimental effects to cellular metabolism and HM cations are typically coordinated by up to four cysteine units [79,92]. MTs are involved in the transport of both essential and non-essential HMs within the cytosol [93] and have been reported to chelate HMs such as Cd, zinc and Cu [79,94,95]. Length and cysteine content of MTs can be taxa specific; for example, most plant MTs are shorter (50–90 AA) and exhibit a lower cysteine content (15–25%) than MTs from other taxa, whereas a higher cysteine contents (> 25%) and a variable length has been observed for fungi (30–70 AA), Metazoa (40–80 AA) and ciliates (100–160 AA) [95]. With the exception of ciliates, for which MTs have been studied in detail [96,97,98], little information is available on MTs from protists. The number of both sequenced genes and scientific publications related to protistan MTs has been reported to be highly underrepresented with respect to the total MTs [85]. MTs have been characterised only in five microalgal genera (*Aureococcus*, *Symbiodinium*, *Thalassiosira*, *Ostreococcus*, *Chlorella* and *Nannochloropsis*) [85]; a recent survey of genomic and transcriptomic databases allowed the prediction of 18 novel potential MTs in microalgae, mostly affiliated to diatoms and dinoflagellates [99]. MTs are also likely to play a role as radical scavengers, protecting cells from oxidative stress [79,100].

## 7. Synergistic Effect of Cu and Other Pollutants

In some microalgae, Cu seems to share transport systems with other metals. Chen and co-workers, for example, described a relation between Pb and Cu internalization in *Chlamydomonas reinhardtii*, that varied depending on the concentration range of both metals, and identified two Cu transport systems, a high-affinity-low-capacity system and a low-affinity-high-capacity one, suggesting that the latter may be used also by Pb to enter the cell [101]. Competition experiments for Pb and Cu in *C. reinhardtii* confirmed that Pb shares with Cu its transport system; in fact, a reciprocal effect between Pb and Cu was observed in the form of a significant decrease in Pb internalization in microalgal cells (87%) in the presence of Cu excess (50 times Pb concentration); the opposite was also true: an excess of Pb in the media led to a decrease in Cu internalization. [102].

Experiments conducted on microalgae exposed to combined pollution of arsenic (As) and Cu seem to indicate that Cu(II) can promote the processes of absorption and speciation of arsenic. Indeed, if compared with exposure to As(V) alone, the simultaneous presence of Cu(II) and As(V) resulted in an enhanced algal ability to absorb and transform As(V). Furthermore, under As-Cu co-exposure, more monomethylarsonous acid (MMA) and dimethylarsinous acid (DMA), volatile organic arsenic compounds, were produced, suggesting an intensification in the algal detoxification mechanism that converts inorganic As to organic As [103].

Cu toxicity can be also influenced by non-HM pollutants. Zhu et. al., for example, performed growth inhibition tests to study combined toxicities of Cu nanoparticles (nano-Cu) and microplastics (MPs) on the diatom *S. costatum*, finding that co-existence with MPs reduced Cu nanoparticles toxicity. This was due to adsorption of Cu^2+^ on microplastic and aggregation between Cu nanoparticles and microplastic, as confirmed by SEM, that resulted in attenuation of Cu toxicity [104]. In a study by Wan et al., on the effect of polystyrene micro- and nanoplastics (MPs/NPs) on Cu toxicity on two freshwater microalgae, *Chlorella* sp. and *P. subcapitata*, they showed that in both microalgae, although sensitive to Cu and tolerant to MPs/NPs, the latter increased Cu toxicity of Cu at EC_50_ in chronic exposure [105]. These studies show that microalgal response to Cu pollution is influenced by several variables, which could also be unpredictable in complex systems as natural environments. However, we believe that information regarding competition experiments among different metals are of particular relevance, since they provide precious information about eventual inhibition or reduction in Cu transport inside cells. We consider these studies as a preliminary step for further experiments to be performed in more complex systems (e.g., environments characterised by high concentrations of more than two metals and/or other pollutants).

## 8. Microalgal Potential for HM Removal, with a Special Focus on Cu: Possibilities and Constraints

In the last decades, microalgae have been regarded as suitable microorganisms for the sequestration of both air and aquatic pollutants. Microalgae show, indeed, good biofixation rates of carbon dioxide [106] and nitric oxides [107], suggesting strategies to improve both algal growth and sequestration of flue gas components in industrial areas. Microalgae are, indeed, suitable candidates for bioremediation because of their high surface-to-volume ratio due to their small size and a subsequent high sorption and bioaccumulation capacities.

Regarding the exploitation of microalgae to remove aquatic contaminants, these microorganisms have shown good skills towards antibiotic [108] and perfluorinated compounds [109], and in wastewater treatments for their ability to use inorganic and organic compounds as nourishment [110,111,112]. However, the most relevant feature is, in our opinion, the capacity of removing persistent pollutants such as heavy metals. In this review article, we focused our attention on the potential of microalgae for Cu removal for two main reasons: (1) this metal is released from several anthropogenic sources, such as fertilisers, pesticides, antifouling paints and some industrial discharges [113] and is thus widespread in aquatic environments, and (2) the potential of microalgae for Cu removal is well known, since this metal is essential for their growth, metabolism and enzyme activities at low concentrations [45] and can be inhibited only in the presence of high Cu levels [114].

Some phycoremediation systems which foresee the employment of microalgae are already in use: they are based on the exploitation of specific microorganisms or a consortium of different organisms and/or microorganisms for pollutant removal, and foresee the employment of natural or synthetic matrixes. The immobilisation of aquatic organisms, indeed, allows to create a more efficient biosorption system that avoids harvesting costs. For example, the AlgaSORB sorption process was developed using dead algal cells of the freshwater species *C. vulgaris* immobilized in a silica gel polymer and it is capable of efficiently removing metallic ions from aqueous solutions [94,115]. Another biosorbent, the BIO-FIX system, is based on a combination of different algae and other organisms, such as yeasts, bacteria and seaweeds immobilized in a high-density polysulfone matrix [94,116]. A final example includes a series of biosorbents produced by Canadian company B. V. SORBEX; they are based on different types of biomaterials, including microalgae (*C. vulgaris*) and five macroalgal species and are effective over a wide range of pH values and are suitable for the removal of a plethora of metal ions [94,117]. The exploitation of dead organisms has a weak point, i.e., the inability of non-viable cells to internalise HMs after their adsorption onto the cell wall that limits the risk of the release of pollutants in the aquatic environment. On the other hand, the main advantage of employing non-living biomasses is the possibility to re-use several times these biosorbents after removing the metals through some desorption cycles with washing agents.

Recently, researchers have moved from the concept of axenic cultures to the study of the entire microbial community for biotechnological applications. Microorganisms, including bacteria, microalgae, fungi, archaea and viruses co-exist in a living complex environment establishing dynamic interactions to cooperate and survive. In particular, it has been demonstrated that microalgae and bacteria can benefit for each other through a mutualism relation especially based on the exchange of nutrients and other signals. As a matter of fact, microalgae can supply oxygen through photosynthesis to the bacteria which, in exchange, promote microalgal growth producing CO_2_ and other metabolites by decomposing organic matter [118,119]. This dual system has been successfully applied for the removal of nutrients present in wastewaters, achieving for example more than 80% reduction in total dissolved nitrogen and phosphorus (TDP) when cultivating together *C. vulgaris* and *Bacillus licheniformis* [120]. In other cases, the combination of the green alga *Auxenochlorella protothecoides*, with *Escherichia coli* under mixotrophic conditions not only led to nutrients removal, but it was also associated with a significantly algal biomass growth and increased lipids content for biofuel production [121]. These results are also in agreement with recent studies where the combination of *Tetradesmus obliquus* and the bacterium *Variovorax paradoxus* in wastewaters resulted in higher microalgal growth, efficient nutrient removal, reduced COD and increased lipids and pigments [122]. Moreover, nutrients reduction the use of mixed cultures increased the cost-efficiency of biogas production in an outdoors algal pond demonstrating both the relevant environmental and economic impact of the co-cultivation approach [123]. These results, combined with the continuous advancements about the molecular and biochemical mechanisms which regulate the microbial interactions, will encourage the application of microalgae and bacteria consortia in other fields, including the bioremediation of heavy metals. Some studies already support this trend. Loutseti and co-workers generated a biofilter composed by a mixture of dried micro-algal and bacterial biomass for removing more than 95% of heavy metals, specifically Cu and Cd, from electroplating wastes. Interestingly, the authors produced the necessary biomass using as main nutrient source the municipal wastewaters, making the bioremediation process more sustainable [124]. The importance of microalgae associated bacteria in bioremediation processes has been demonstrated by co-cultivating *C. vulgaris* and *Enterobacter* sp. for the decolorization and removal of heavy metals from textile wastewater. In that study the presence of the bacteria stimulated the microalga growth with a consequent reduction of above 70% of Cu, chromium, Cd and Pb [125]. Although the development of a proper model to exploit the full potential of microalgae-bacteria consortium is still far to be established [126], the use of Microalga Growth-Promoting Bacteria (MGPB), as defined in different publications, seems to be a promising strategy to increase algal biomass and reduce heavy metals, or other pollutants, concentration [127]. In support, Yu et al., reported a more efficient Cd accumulation when a higher bacteria concentration was added to the mixed system composed by *Chlorella salina* and *Bacillus subtilis* [128]. An efficient treatment of Cu mine wastewater was obtained using immobilized sulphate-reducing bacteria beads with microalgae biomass as the sole nutrient sources [129]. Sustainability and cost-efficiency are the drivers which are leading to the application of complex systems instead of single cultures, and microalgae-bacteria consortium represent a promising solution for several biotechnological applications.

The above mentioned examples clearly demonstrate that co-cultivation of microalgae with other aquatic microorganisms (especially bacteria) could be considered as a suitable strategy for a sustainable removal of HMs, including Cu, from freshwater and saline environments. Of course, the use of allochthonous living species limits the use of microalgae to ex situ techniques of HM removal to avoid alterations in biodiversity. For a direct HM removal, it would be desirable to employ autochthonous algae which are able to sustain a positive growth also in the presence relatively high HM amounts, able to adsorb or compartmentalize them, eventually exploiting the synergistic effect of different kinds of microorganisms.

## 9. Conclusions

Several studies concerning Cu effects on microalgae have been here reviewed. While at trace concentrations this HM is essential for all organisms, anthropogenic activities constitute an ever-increasing source for its release and build-up into the environment, where, if above certain thresholds, can become toxic for ecosystems. Microalgae are one of the first groups to be visibly affected by HM pollution, with detectable signs of Cu stress ranging from cells size and shape alterations to photosynthesis deregulation and oxidative stress generation, and could be therefore used as bio-indicators to monitor early signs of this HM stress effect on ecosystems. In addition, some microalgal species can tolerate Cu up to environmentally relevant concentrations and, in some cases, even higher. Moreover, even though the literature on the subject is still limited, it seems that they are able to remove Cu from the environment due to their ability to adsorb and/or actively transport the toxicant inside the cell, and are thus ideal candidates for bioremediation approaches. In light of this, further studies specifically aimed at elucidating Cu removal rates in several microalgal species are needed to exploit their potential for this kind of applications.

## Data Availability

Not applicable.

## References

[1] Ali H., Khan E. (2018). What are heavy metals? Long-standing controversy over the scientific use of the term ‘heavy metals’—Proposal of a comprehensive definition. Toxicol. Environ. Chem..

[2] Tchounwou P.B., Yedjou C.G., Patlolla A.K., Sutton D.J. (2012). Heavy metal toxicity and the environment. Exp. Suppl..

[3] He Z.L.L., Yang X.E., Stoffella P.J. (2005). Trace elements in agroecosystems and impacts on the environment. J. Trace Elem. Med. Biol..

[4] Clark R.B., Frid C., Attrill M. (2001). Marine Pollution.

[5] Fu Z.S., Xi S.H. (2020). The effects of heavy metals on human metabolism. Toxicol. Mech. Methods.

[6] Rial D., Beiras R. (2012). Prospective ecological risk assessment of sediment resuspension in an estuary. J. Environ. Monit..

[7] Fichet D., Radenac G., Miramand P. (1998). Experimental studies of impacts of harbour sediments resuspension to marine invertebrates larvae: Bioavailability of Cd, Cu, Pb and Zn and toxicity. Mar. Pollut. Bull..

[8] Shull D., Cochran J.K., Bokuniewicz H.J., Yager P.L. (2019). Bioturbation. Encyclopedia of Ocean Sciences, Vol 3: Ocean Dynamics.

[9] Cordoba-Tovar L., Marrugo-Negrete J., Baron P.R., Diez S. (2022). Drivers of biomagnification of Hg, As and Se in aquatic food webs: A review. Environ. Res..

[10] Wu P.P., Zakem E.J., Dutkiewicz S., Zhang Y.X. (2020). Biomagnification of Methylmercury in a Marine Plankton Ecosystem. Environ. Sci. Technol..

[11] Xie Q., Gui D., Liu W., Wu Y.P. (2020). Risk for Indo-Pacific humpback dolphins (*Sousa chinensis*) and human health related to the heavy metal levels in fish from the Pearl River Estuary, China. Chemosphere.

[12] Vu C.T., Lin C., Yeh G., Villanueva M.C. (2017). Bioaccumulation and potential sources of heavy metal contamination in fish species in Taiwan: Assessment and possible human health implications. Environ. Sci. Pollut. Res..

[13] Köker L., Aydın F., Gaygusuz Ö., Akçaalan R., Çamur D., İlter H., Ayoğlu F.N., Altın A., Topbaş M., Albay M. (2021). Heavy Metal Concentrations in *Trachurus mediterraneus* and *Merlangius merlangus* Captured from Marmara Sea, Turkey and Associated Health Risks. Environ. Manag..

[14] De Mora S., Fowler S.W., Wyse E., Azemard S. (2004). Distribution of heavy metals in marine bivalves, fish and coastal sediments in the Gulf and Gulf of Oman. Mar. Pollut. Bull..

[15] Culha S.T., Culha M., Karayucel I., Celik M.Y., Isler Y. (2017). Heavy metals in *Mytilus galloprovincialis*, suspended particulate matter and sediment from offshore submerged longline system, Black Sea. Int. J. Environ. Sci. Technol..

[16] Mejdoub Z., Zaid Y., Hmimid F., Kabine M. (2018). Assessment of metals bioaccumulation and bioavailability in mussels *Mytilus galloprovincialis* exposed to outfalls pollution in coastal areas of Casablanca. J. Trace Elem. Med. Biol..

[17] Joksimovic D., Castelli A., Perosevic A., Djurovic D., Stankovic S. (2018). Determination of trace metals in *Mytilus galloprovincialis* along the Boka Kotorska Bay, Montenegrin coast. J. Trace Elem. Med. Biol..

[18] Kouali H., Chaouti A., Achtak H., Elkalay K., Dahbi A. (2022). Trace metal contents in the mussel *Mytilus galloprovincialis* from Atlantic coastal areas in northwestern Morocco: Levels of contamination and assessment of potential risks to human health. Mar. Pollut. Bull..

[19] Mehri A. (2020). Trace Elements in Human Nutrition (II)—An Update. Int. J. Prev. Med..

[20] World Health Organization, International Atomic Energy Agency, Food and Agriculture Organization of the United Nations (1996). Trace Elements in Human Nutrition and Health.

[21] Roveta C., Annibaldi A., Afghan A., Calcinai B., Di Camillo C.G., Gregorin C., Illuminati S., Pulido Mantas T., Truzzi C., Puce S. (2021). Biomonitoring of Heavy Metals: The Unexplored Role of Marine Sessile Taxa. Appl. Sci..

[22] Jovic M., Onjia A., Stankovic S. (2012). Toxic metal health risk by mussel consumption. Environ. Chem. Lett..

[23] Ramirez R. (2013). The gastropod *Osilinus atrata* as a bioindicator of Cd, Cu, Pb and Zn contamination in the coastal waters of the Canary Islands. Chem. Ecol..

[24] Tsang T., Davis C.I., Brady D.C. (2021). Copper biology. Curr. Biol. CB.

[25] Scheiber I., Dringen R., Mercer J.F. (2013). Copper: Effects of deficiency and overload. Met. Ions Life Sci..

[26] Franchitto N., Gandia-Mailly P., Georges B., Galinier A., Telmon N., Ducasse J.L., Rouge D. (2008). Acute copper sulphate poisoning: A case report and literature review. Resuscitation.

[27] Amuda O.S., Alade A.O., Hung Y.T., Wang L.K., Wang M.H.S. (2016). Toxicity, Sources, and Control of Copper (Cu), Zinc (Zn), Molybdenum (Mo), Silver (Ag), and Rare Earth Elements in the Environment. Civ. Environ. Eng. Fac. Publ..

[28] Zolkefli N., Sharuddin S.S., Yusoff M.Z.M., Hassan M.A., Maeda T., Ramli N. (2020). A Review of Current and Emerging Approaches for Water Pollution Monitoring. Water.

[29] Nowicka B. (2022). Heavy metal-induced stress in eukaryotic algae-mechanisms of heavy metal toxicity and tolerance with particular emphasis on oxidative stress in exposed cells and the role of antioxidant response. Environ. Sci. Pollut. Res..

[30] Marella T.K., Saxena A., Tiwari A. (2020). Diatom mediated heavy metal remediation: A review. Bioresour. Technol..

[31] Danouche M., El Ghachtouli N., El Arroussi H. (2021). Phycoremediation mechanisms of heavy metals using living green microalgae: Physicochemical and molecular approaches for enhancing selectivity and removal capacity. Heliyon.

[32] Goswami R.K., Agrawal K., Shah M.P., Verma P. (2021). Bioremediation of heavy metals from wastewater: A current perspective on microalgae-based future. Lett. Appl. Microbiol..

[33] Crichton R.R., Pierre J.L. (2001). Old iron, young copper: From Mars to Venus. Biometals.

[34] Quigg A., Borowitzka M., Beardall J., Raven J. (2016). Micronutrients. The Physiology of Microalgae, Vol 6: Developments in Applied Phycology.

[35] De la Cerda B., Castielli O., Durán R.V., Navarro J.A., Hervás M., De la Rosa M.A. (2007). A proteomic approach to iron and copper homeostasis in cyanobacteria. Brief Funct. Genom. Proteom..

[36] Kropat J., Gallaher S.D., Urzica E.I., Nakamoto S.S., Strenkert D., Tottey S., Mason A.Z., Merchant S.S. (2015). Copper economy in *Chlamydomonas*: Prioritized allocation and reallocation of copper to respiration vs. photosynthesis. Proc. Natl. Acad. Sci. USA.

[37] Wang X.J., Cao W., Du H., Liu W.H., Li P. (2021). Increasing Temperature Alters the Effects of Extracellular Copper on *Thalassiosira Pseudonana* Physiology and Transcription. J. Mar. Sci. Eng..

[38] Maldonado M.T., Allen A.E., Chong J.S., Lin K., Leus D., Karpenko N., Harris S.L. (2006). Copper-dependent iron transport in coastal and oceanic diatoms. Limnol. Oceanogr..

[39] La Fontaine S., Quinn J.M., Nakamoto S.S., Page M.D., Gohre V., Mosely J.L., Kropat J., Merchant S. (2002). Copper-dependent iron assimilation pathway in the model photosynthetic eukaryote *Chlamydomonas reinhardtii*. Eukaryot. Cell.

[40] Guo J., Lapi S., Ruth T.J., Maldonado M.T. (2012). The effects of iron and copper availability on the copper stoichiometry on marine phytoplankton. J. Phycol..

[41] Annett A.L., Lapi S., Ruth T.J., Maldonado M.T. (2008). The effects of Cu and Fe availability on the growth and Cu : C ratios of marine diatoms. Limnol. Oceanogr..

[42] Peers G., Quesnel S.A., Price N.M. (2005). Copper requirements for iron acquisition and growth of coastal and oceanic diatoms. Limnol. Oceanogr..

[43] Silva J.C., Echeveste P., Lombardi A.T. (2018). Higher biomolecules yield in phytoplankton under copper exposure. Ecotoxicol. Environ. Saf..

[44] El Agawany N., Kaamoush M., El-Zeiny A., Ahmed M. (2021). Effect of heavy metals on protein content of marine unicellular green alga *Dunaliella tertiolecta*. Environ. Monit. Assess..

[45] Kong L., Price N.M. (2020). Identification of copper-regulated proteins in an oceanic diatom, *Thalassiosira oceanica* 1005. Met. Integr. Biomet. Sci..

[46] Rocha G.S., Parrish C.C., Espindola E.L.G. (2021). Effects of copper on photosynthetic and physiological parameters of a freshwater microalga (Chlorophyceae). Algal Res. Biomass Biofuels Bioprod..

[47] Torres M.A., Barros M.P., Campos S.C.G., Pinto E., Rajamani S., Sayre R.T., Colepicolo P. (2008). Biochemical biomarkers in algae and marine pollution: A review. Ecotoxicol. Environ. Saf..

[48] Das N., Vimala R., Karthika P. (2008). Biosorption of heavy metals—An overview. Indian J. Biotechnol..

[49] He J.S., Chen J.P. (2014). A comprehensive review on biosorption of heavy metals by algal biomass: Materials, performances, chemistry, and modeling simulation tools. Bioresour. Technol..

[50] Levy J.L., Angel B.M., Stauber J.L., Poon W.L., Simpson S.L., Cheng S.H., Jolley D.F. (2008). Uptake and internalisation of copper by three marine microalgae: Comparison of copper-sensitive and copper-tolerant species. Aquat. Toxicol..

[51] Narula P., Mahajan A., Gurnani C., Kumar V., Mukhija S. (2015). Microalgae as an Indispensable Tool against Heavy Metals Toxicity to Plants: A Review. Int. J. Pharm. Sci. Rev. Res..

[52] Quigg A., Reinfelder J.R., Fisher N.S. (2006). Copper uptake kinetics in diverse marine phytoplankton. Limnol. Oceanogr..

[53] Croot P.L., Karlson B., van Elteren J.T., Kroon J.J. (2003). Uptake and efflux of Cu-64 by the marine cyanobacterium *Synechococcus* (WH7803). Limnol. Oceanogr..

[54] Blaby-Haas C.E., Merchant S.S. (2012). The ins and outs of algal metal transport. Biochim. Biophys. Acta.

[55] Nassiri Y., Mansot J.L., Wery J., GinsburgerVogel T., Amiard J.C. (1997). Ultrastructural and electron energy loss spectroscopy studies of sequestration mechanisms of Cd and Cu in the marine diatom *Skeletonema costatum*. Arch. Environ. Contam. Toxicol..

[56] Adams M.S., Dillon C.T., Vogt S., Lai B., Stauber J., Jolley D.F. (2016). Copper Uptake, Intracellular Localization, and Speciation in Marine Microalgae Measured by Synchrotron Radiation X-ray Fluorescence and Absorption Microspectroscopy. Environ. Sci. Technol..

[57] Croot P.L., Moffett J.W., Brand L.E. (2000). Production of extracellular Cu complexing ligands by eucaryotic phytoplankton in response to Cu stress. Limnol. Oceanogr..

[58] Martinez-Macias M.D., Correa-Murrieta M.A., Villegas-Peralta Y., Devora-Isiordia G.E., Alvarez-Sanchez J., Saldivar-Cabrales J., Sanchez-Duarte R.G. (2019). Uptake of copper from acid mine drainage by the microalgae *Nannochloropsis oculata*. Environ. Sci. Pollut. Res..

[59] Hamed S.M., Selim S., Klock G., AbdElgawad H. (2017). Sensitivity of two green microalgae to copper stress: Growth, oxidative and antioxidants analyses. Ecotoxicol. Environ. Saf..

[60] Gong A.P., Gu W.M., Zhao Z.Y., Shao Y.N. (2019). Identification of heavy metal by testing microalgae using confocal Raman microspectroscopy technology. Appl. Opt..

[61] Manimaran K., Karthikeyan P., Ashokkumar S., Prabu V.A., Sampathkumar P. (2012). Effect of Copper on Growth and Enzyme Activities of Marine Diatom, *Odontella mobiliensis*. Bull. Environ. Contam. Toxicol..

[62] Purbonegoro T., Suratno, Puspitasari R., Husna N.A. Toxicity of copper on the growth of marine microalgae Pavlova sp. and its chlorophyll-a. Proceedings of the 1st Global Colloquium on GeoSciences and Engineering (GCGE).

[63] Neethu K.V., Saranya K.S., Krishna N.G.A., Praved P.H., Aneesh B.P., Nandan S.B., Marigoudar S.R. (2021). Toxicity of copper on marine diatoms, *Chaetoceros calcitrans* and *Nitzchia closterium* from Cochin estuary, India. Ecotoxicology.

[64] Levy J.L., Stauber J.L., Jolley D.F. (2007). Sensitivity of marine microalgae to copper: The effect of biotic factors on copper adsorption and toxicity. Sci. Total Environ..

[65] Buayam N., Davey M.P., Smith A.G., Pumas C. (2019). Effects of Copper and pH on the Growth and Physiology of *Desmodesmus* sp. AARLG074. Metabolites.

[66] Melegari S.P., Perreault F., Costa R.H.R., Popovic R., Matias W.G. (2013). Evaluation of toxicity and oxidative stress induced by copper oxide nanoparticles in the green alga *Chlamydomonas reinhardtii*. Aquat. Toxicol..

[67] Li M., Hu C.W., Zhu Q., Chen L., Kong Z.M., Liu Z.L. (2006). Copper and zinc induction of lipid peroxidation and effects on antioxidant enzyme activities in the microalga *Pavlova viridis* (Prymnesiophyceae). Chemosphere.

[68] Imlay J.A. (2003). Pathways of oxidative damage. Annu. Rev. Microbiol..

[69] Pinto E., Sigaud-Kutner T.C.S., Leitao M.A.S., Okamoto O.K., Morse D., Colepicolo P. (2003). Heavy metal-induced oxidative stress in algae. J. Phycol..

[70] Knauert S., Knauer K. (2008). The role of reactive oxygen species in copper toxicity to two freshwater green algae^1^. J. Phycol..

[71] Morelli E., Scarano G. (2004). Copper-induced changes of non-protein thiols and antioxidant enzymes in the marine microalga *Phaeodactylum tricomutum*. Plant Sci..

[72] Sabatini S.E., Juarez A.B., Eppis M.R., Bianchi L., Luquet C.M., de Molina M.D.R. (2009). Oxidative stress and antioxidant defenses in two green microalgae exposed to copper. Ecotoxicol. Environ. Saf..

[73] Wang L., Huang X.L., Sun W.L., Too H.Z., Laserna A.K.C., Li S.F.Y. (2020). A global metabolomic insight into the oxidative stress and membrane damage of copper oxide nanoparticles and microparticles on microalga *Chlorella vulgaris*. Environ. Pollut..

[74] Kwon D.H., Cha H.-J., Lee H., Hong S.-H., Park C., Park S.-H., Kim G.-Y., Kim S., Kim H.-S., Hwang H.-J. (2019). Protective Effect of Glutathione against Oxidative Stress-induced Cytotoxicity in RAW 264.7 Macrophages through Activating the Nuclear Factor Erythroid 2-Related Factor-2/Heme Oxygenase-1 Pathway. Antioxidants.

[75] Meister A., Anderson M.E. (1983). Glutathione. Annu. Rev. Biochem..

[76] Ahner B.A., Wei L.P., Oleson J.R., Ogura N. (2002). Glutathione and other low molecular weight thiols in marine phytoplankton under metal stress. Mar. Ecol.-Prog. Ser..

[77] Leal M.F.C., Vasconcelos M., van den Berg C.M.G. (1999). Copper-induced release of complexing ligands similar to thiols by *Emiliania huxleyi* in seawater cultures. Limnol. Oceanogr..

[78] Musgrave W.B., Yi H., Kline D., Cameron J.C., Wignes J., Dey S., Pakrasi H.B., Jez J.M. (2013). Probing the origins of glutathione biosynthesis through biochemical analysis of glutamate-cysteine ligase and glutathione synthetase from a model photosynthetic prokaryote. Biochem. J..

[79] Cobbett C., Goldsbrough P. (2002). Phytochelatins and metallothioneins: Roles in Heavy Metal Detoxification and Homeostasis. Annu. Rev. Plant Biol..

[80] Hirata K., Tsuji N., Miyamoto K. (2005). Biosynthetic regulation of phytochelatins, heavy metal-binding peptides. J. Biosci. Bioeng..

[81] Howe G., Merchant S. (1992). Heavy metal-activated synthesis of peptides in *Chlamydomonas reinhardtii*. Plant Physiol..

[82] Peng K.-T., Zheng C.-N., Xue J., Chen X.-Y., Yang W.-D., Liu J.-S., Bai W., Li H.-Y. (2014). Delta 5 Fatty Acid Desaturase Upregulates the Synthesis of Polyunsaturated Fatty Acids in the Marine Diatom *Phaeodactylum tricornutum*. J. Agric. Food Chem..

[83] Aviles C., Loza-Tavera H., Terry N., Moreno-Sanchez R. (2003). Mercury pretreatment selects an enhanced cadmium-accumulating phenotype in *Euglena gracilis*. Arch. Microbiol..

[84] Mendoza-Cozatl D., Devars S., Loza-Tavera H., Moreno-Sanchez R. (2002). Cadmium accumulation in the chloroplast of *Euglena gracilis*. Physiol. Plant..

[85] Balzano S., Sardo A., Blasio M., Chahine T.B., Dell’Anno F., Sansone C., Brunet C. (2020). Microalgal Metallothioneins and Phytochelatins and Their Potential Use in Bioremediation. Front. Microbiol..

[86] Ahner B.A., Kong S., Morel F.M.M. (1995). Phytochelatin production in marine algae. 1. An interspecific comparison. Limnol. Oceanogr..

[87] Ahner B.A., Morel F.M.M. (1995). Phytochelatin production in marine algae. 2. Induction by various metals. Limnol. Oceanogr..

[88] Tsuji N., Hirayanagi N., Iwabe O., Namba T., Tagawa M., Miyamoto S., Miyasaka H., Takagi M., Hirata K., Miyamoto K. (2003). Regulation of phytochelatin synthesis by zinc and cadmium in marine green alga, *Dunaliella tertiolecta*. Phytochemistry.

[89] Kalinowska R., Pawlik-Skowrońska B. (2010). Response of two terrestrial green microalgae (Chlorophyta, Trebouxiophyceae) isolated from Cu-rich and unpolluted soils to copper stress. Environ. Pollut..

[90] Zhang W., Tan N.G., Fu B., Li S.F. (2015). Metallomics and NMR-based metabolomics of *Chlorella* sp. reveal the synergistic role of copper and cadmium in multi-metal toxicity and oxidative stress. Met. Integr. Biomet. Sci..

[91] Ziller A., Fraissinet-Tachet L. (2018). Metallothionein diversity and distribution in the tree of life: A multifunctional protein. Met. Integr. Biomet. Sci..

[92] Leszczyszyn O.I., Imam H.T., Blindauer C.A. (2013). Diversity and distribution of plant metallothioneins: A review of structure, properties and functions. Met. Integr. Biomet. Sci..

[93] Capdevila M., Atrian S. (2011). Metallothionein protein evolution: A miniassay. J. Biol. Inorg. Chem..

[94] Kumar K.S., Dahms H.U., Won E.J., Lee J.S., Shin K.H. (2015). Microalgae—A promising tool for heavy metal remediation. Ecotoxicol. Environ. Saf..

[95] Ziller A., Yadav R.K., Capdevila M., Reddy M.S., Vallon L., Marmeisse R., Atrian S., Palacios O., Fraissinet-Tachet L. (2017). Metagenomics analysis reveals a new metallothionein family: Sequence and metal-binding features of new environmental cysteine-rich proteins. J. Inorg. Biochem..

[96] Gutierrez J.C., Amaro F., Diaz S., de Francisco P., Cubas L.L., Martin-Gonzalez A. (2011). Ciliate metallothioneins: Unique microbial eukaryotic heavy-metal-binder molecules. J. Biol. Inorg. Chem..

[97] Gutiérrez J.-C., de Francisco P., Amaro F., Díaz S., Martín-González A., Das S., Dash H.R. (2019). Chapter 22—Structural and Functional Diversity of Microbial Metallothionein Genes. Microbial Diversity in the Genomic Era.

[98] Zahid M.T., Shakoori F.R., Zulfiqar S., Al-Ghanim K.A., Shakoori A.R. (2018). Growth Characteristics, Metal Uptake and Expression Analysis of Copper Metallothionein in a Newly Reported Ciliate, Tetrahymena farahensis. Pak. J. Zool..

[99] Balzano S., Sardo A. (2022). Bioinformatic prediction of putative metallothioneins in non-ciliate protists. Biol. Lett..

[100] Ruttkay-Nedecky B., Nejdl L., Gumulec J., Zitka O., Masarik M., Eckschlager T., Stiborova M., Adam V., Kizek R. (2013). The Role of Metallothionein in Oxidative Stress. Int. J. Mol. Sci..

[101] Chen Z.Z., Zhu L., Wilkinson K.J. (2010). Validation of the Biotic Ligand Model in Metal Mixtures: Bioaccumulation of Lead and Copper. Environ. Sci. Technol..

[102] Sanchez-Marin P., Fortin C., Campbell P.G.C. (2014). Lead (Pb) and copper (Cu) share a common uptake transporter in the unicellular alga *Chlamydomonas reinhardtii*. Biometals.

[103] Huang Z.Q., Chen B.Y., Zhang J., Yang C.L., Wang J., Song F., Li S.G. (2021). Absorption and speciation of arsenic by microalgae under arsenic-copper Co-exposure. Ecotoxicol. Environ. Saf..

[104] Zhu X., Zhao W., Chen X., Zhao T., Tan L., Wang J. (2020). Growth inhibition of the microalgae *Skeletonema costatum* under copper nanoparticles with microplastic exposure. Mar. Environ. Res.

[105] Wan J.K., Chu W.L., Kok Y.Y., Lee C.S. (2021). Influence of polystyrene microplastic and nanoplastic on copper toxicity in two freshwater microalgae. Environ. Sci. Pollut. Res. Int..

[106] Chen B.Y., Lee C.H., Hsueh C.C. Dose-response assessment upon CO_2_ tolerance of indigenous microalgal isolates for biofuel production. Proceedings of the 6th International Conference on Applied Energy (ICAE).

[107] Nagase H., Yoshihara K.-i., Eguchi K., Okamoto Y., Murasaki S., Yamashita R., Hirata K., Miyamoto K. (2001). Uptake pathway and continuous removal of nitric oxide from flue gas using microalgae. Biochem. Eng. J..

[108] Mojiri A., Baharlooeian M., Zahed M.A. (2021). The Potential of *Chaetoceros muelleri* in Bioremediation of Antibiotics: Performance and Optimization. Int. J. Environ. Res. Public Health.

[109] Albert K., Hsieh P.Y., Chen T.H., Hou C.H., Hsu H.Y. (2020). Diatom-assisted biomicroreactor targeting the complete removal of perfluorinated compounds. J. Hazard. Mater..

[110] Minggat E., Roseli W., Tanaka Y. (2021). Nutrient Absorption and Biomass Production by the Marine Diatom *Chaetoceros muelleri*: Effects of Temperature, Salinity, Photoperiod, and Light Intensity. J. Ecol. Eng..

[111] Gao F., Li C., Yang Z.H., Zeng G.M., Mu J., Liu M., Cui W. (2016). Removal of nutrients, organic matter, and metal from domestic secondary effluent through microalgae cultivation in a membrane photobioreactor. J. Chem. Technol. Biotechnol..

[112] Scarponi P., Ghirardini A.M.V., Bravi M., Cavinato C. (2021). Evaluation of *Chlorella vulgaris* and *Scenedesmus obliquus* growth on pretreated organic solid waste digestate. Waste Manag..

[113] Stauber J.L., Davies C.M. (2000). Use and limitations of microbial bioassays for assessing copper bioavailability in the aquatic environment. Environ. Rev..

[114] Wang L., Zheng B. (2008). Toxic effects of fluoranthene and copper on marine diatom *Phaeodactylum tricornutum*. J. Environ. Sci..

[115] Oliveira R.C., Palmieri M.C., Garcia O. (2011). Biosorption of Metals: State of the Art, General Features, and Potential Applications for Environmental and Technological Processes. Progress in Biomass and Bioenergy Production.

[116] Vijayaraghavan K., Yun Y.S. (2008). Bacterial biosorbents and biosorption. Biotechnol. Adv..

[117] Wang J.L., Chen C. (2009). Biosorbents for heavy metals removal and their future. Biotechnol. Adv..

[118] Zhang B., Li W., Guo Y., Zhang Z., Shi W., Cui F., Lens P.N.L., Tay J.-H. (2020). Microalgal-bacterial consortia: From interspecies interactions to biotechnological applications. Renew. Sustain. Energy Rev..

[119] Fallahi A., Rezvani F., Asgharnejad H., Khorshidi Nazloo E., Hajinajaf N., Higgins B. (2021). Interactions of microalgae-bacteria consortia for nutrient removal from wastewater: A review. Chemosphere.

[120] Ji X., Jiang M., Zhang J., Jiang X., Zheng Z. (2018). The interactions of algae-bacteria symbiotic system and its effects on nutrients removal from synthetic wastewater. Bioresour. Technol..

[121] Higgins B.T., Gennity I., Samra S., Kind T., Fiehn O., VanderGheynst J.S. (2016). Cofactor symbiosis for enhanced algal growth, biofuel production, and wastewater treatment. Algal Res..

[122] Perera I.A., Abinandan S., Panneerselvan L., Subashchandrabose S.R., Venkateswarlu K., Naidu R., Megharaj M. (2022). Co-culturing of microalgae and bacteria in real wastewaters alters indigenous bacterial communities enhancing effluent bioremediation. Algal Res..

[123] Marín D., Posadas E., Cano P., Pérez V., Lebrero R., Muñoz R. (2018). Influence of the seasonal variation of environmental conditions on biogas upgrading in an outdoors pilot scale high rate algal pond. Bioresour. Technol..

[124] Loutseti S., Danielidis D.B., Economou-Amilli A., Katsaros C., Santas R., Santas P. (2009). The application of a micro-algal/bacterial biofilter for the detoxification of copper and cadmium metal wastes. Bioresour. Technol..

[125] Mubashar M., Naveed M., Mustafa A., Ashraf S., Shehzad Baig K., Alamri S., Siddiqui M.H., Zabochnicka-Świątek M., Szota M., Kalaji H.M. (2020). Experimental Investigation of *Chlorella vulgaris* and *Enterobacter* sp. MN17 for Decolorization and Removal of Heavy Metals from Textile Wastewater. Water.

[126] Zuñiga C., Li T., Guarnieri M.T., Jenkins J.P., Li C.-T., Bingol K., Kim Y.-M., Betenbaugh M.J., Zengler K. (2020). Synthetic microbial communities of heterotrophs and phototrophs facilitate sustainable growth. Nat. Commun..

[127] Palacios O.A., López B.R., de-Bashan L.E. (2022). Microalga Growth-Promoting Bacteria (MGPB): A formal term proposed for beneficial bacteria involved in microalgal–bacterial interactions. Algal Res..

[128] Yu Q., Li P., Li B., Zhang C., Zhang C., Ge Y. (2022). Effects of algal–bacterial ratio on the growth and cadmium accumulation of *Chlorella salina*–*Bacillus subtilis* consortia. J. Basic Microbiol..

[129] Li Y., Yang X., Geng B. (2018). Preparation of Immobilized Sulfate-Reducing Bacteria-Microalgae Beads for Effective Bioremediation of Copper-Containing Wastewater. Water Air Soil Pollut..

